# Clinical Diabetic Peripheral Neuropathy: Can It Be Reversed? Arguments for and Against From a NEUROdiab Debate

**DOI:** 10.1111/jns.70124

**Published:** 2026-05-17

**Authors:** Gordon Sloan, Shazli Azmi, Stephanie Eid, Maryam Ferdousi, Johan Røikjer, Zoltan Kender, Luca D'Onofrio, Eleni Karlafti, Brian Callaghan, Bruce A. Perkins

**Affiliations:** ^1^ Division of Clinical Medicine University of Sheffield Sheffield UK; ^2^ Centre for Diabetes, Endocrinology and Metabolism Manchester University NHS Foundation Trust Manchester UK; ^3^ Division of Cardiovascular Sciences, Faculty of Biology, Medicine and Health The University of Manchester Manchester UK; ^4^ Department of Neurology University of Michigan Ann Arbor Michigan USA; ^5^ Department of Anatomy, Cell Biology, and Physiological Sciences, Faculty of Medicine and Medical Center American University of Beirut Beirut Lebanon; ^6^ Manchester University NHS Foundation Trust Manchester UK; ^7^ Steno Diabetes Center North Denmark Aalborg University Hospital Aalborg Denmark; ^8^ Department of Clinical Medicine Aalborg University Aalborg Denmark; ^9^ Clinic for Endocrinology, Diabetology, Metabolic Diseases and Clinical Chemistry (Internal Medicine 1) Heidelberg University Hospital Heidelberg Germany; ^10^ German Center of Diabetes Research (DZD) Neuherberg Germany; ^11^ Diabetology Unit AOU Policlinico Umberto I Rome Italy; ^12^ Emergency Department University General Hospital of Thessaloniki AHEPA, Aristotle University of Thessaloniki Thessaloniki Greece; ^13^ University of Toronto Toronto Ontario Canada

**Keywords:** diabetes, diabetic neuropathy, peripheral neuropathy

## Abstract

Diabetic peripheral neuropathy (DPN) is a prevalent and disabling complication of diabetes, yet whether established clinical DPN is reversible remains debated. At the 35th Annual Meeting of NEUROdiab, a formal debate examined arguments ‘for’ and ‘against’ the proposition that clinical DPN can be reversed. This review summarises both perspectives and considers their implications for clinical practice and future research. Evidence supporting the reversibility of clinical DPN draws on four main observations. Therapies for hereditary transthyretin amyloidosis demonstrate that substantial improvement in a progressive length‐dependent neuropathy is biologically achievable, providing a proof‐of‐concept in a different disease. Pancreatic transplantation in Type 1 diabetes leads to measurable improvements in large‐ and small‐fibre indices. Structured lifestyle interventions and bariatric surgery improve intraepidermal nerve fibre density, suggesting that metabolic correction can promote neural repair. Finally, emerging agents such as topical oxybutynin show early promise in improving surrogate outcomes, reinforcing the argument that clinical DPN may be partially reversible. Arguments opposing reversibility emphasise that DPN resembles other microvascular complications, in which early abnormalities may regress but established clinical disease is not reversed. Heterogeneity in diagnostic thresholds, phenotype variability and inconsistent outcome measures complicates defining what constitutes true reversal. Long‐term studies and major trials, including DCCT/EDIC and BARI 2D, demonstrate slowing of progression rather than restoration of normal nerve function. Moreover, numerous mechanistically promising agents have failed in human trials despite strong pre‐clinical results. Collectively, these limitations support the view that established clinical neuropathy remains largely irreversible to date. Overall, the debate underscores that reversibility depends on timing, definitions and outcome thresholds. While early‐stage dysfunction is more likely to be modifiable, complete restoration of established clinical DPN (such as full restoration of protective sensation) remains unproven. Future research should prioritise developing validated biomarkers and standardised endpoints in addition to searching for new therapeutics.

## 1 Introduction

Diabetic peripheral neuropathy (DPN) is one of the most common and disabling complications of diabetes, affecting up to half of individuals with long‐standing disease [1]. It presents with pain, numbness, sensory loss, imbalance, and, in severe cases, foot ulceration and amputation. Its economic burden is substantial, with those affected incurring significantly greater healthcare utilisation and costs compared to those with diabetes alone [2, 3]. DPN is typically diagnosed only after significant nerve damage has occurred [4]. Consequently, therapeutic efforts typically focus on symptomatic relief and complication prevention, rather than restoration of nerve function. Despite this consensus, whether clinical DPN can be reversed remains uncertain. Advances in diabetes management, pancreatic transplantation (PT) and lifestyle interventions have shown some improvement in surrogate markers and some clinical measures of neuropathy [5, 6, 7, 8, 9]. However, long‐term observational studies and randomised trials indicate established DPN is progressive and not reversible [5]. Repeated failures of promising therapies further highlight the difficulty of translating advances into clinical benefit [10, 11, 12]. This ongoing divide between emerging optimism and persistent scepticism highlights the critical need to reassess what ‘reversibility’ truly means within the context of clinical DPN.

Against this backdrop, the 35th Annual Meeting of NEUROdiab (the Diabetic Neuropathy Study Group acting as a reference group for the EASD in matters related to diabetic neuropathy) convened in Bucharest, Romania. The NEUROdiab Youth Committee organised a formal debate entitled: ‘This house believes that clinical diabetic peripheral neuropathy is reversible’. Two leading experts, Professor Brian Callaghan (University of Michigan Medical School, USA) and Professor Bruce Perkins (Toronto General Hospital Research Institute, Canada), presented opposing arguments. This review summarises the key positions from that debate, highlighting evidence for potential reversal, alongside counterarguments emphasising the biological, methodological and clinical barriers to such a claim.

## 2 The Case *for* Clinical DPN Being Reversible

The case for clinical DPN being reversible rests on four main arguments (Table 1): (1) Data from hereditary transthyretin amyloidosis (ATTRv) show that reversal of clinical peripheral neuropathies is possible; (2) PT demonstrates improvement in DPN outcomes; (3) bariatric surgery and lifestyle interventions improve nerve fibre density; (4) novel treatments for DPN show promising early results, providing a proof‐of‐concept for reversal.

**TABLE 1:** Summary of interventional studies reporting improvement in structural or functional measures of peripheral neuropathy (PN).

Study	Evidence level	Sample	Active intervention	Follow‐up	Outcome metrics	Results
Adams et al. 2018	Randomised controlled trial	225 patients with ATTRv (148 patisiran vs. 77 placebo)	Patisiran	18 months	mNIS+7	Mean improvement in neuropathy with patisiran, 56% demonstrating improvement in mNIS+7
Benson et al. 2018	Randomised controlled trial	173 patients with ATTRv (112 inotersen vs. 60 placebo)	Inotersen	15 months	mNIS+7	Slowed progression of neuropathy compared with placebo, with 36% demonstrating improvement in mNIS+7
Boswell et al. 2023	Retrospective cohort study	187 patients with T1DM	SPK	11.3 years	VPT	Significant improvement in VPT, and reduction in % of abnormal VPT results
Azmi et al. 2019	Case–control retrospective	36 patients with T1DM	SPK	36 months	NCS, VPT, CCM, IENFD, symptoms	Improvement in CNFD and CNFL, peroneal nerve conduction velocity and neuropathy symptoms
Boggi et al. 2011	Retrospective cohort study	71 patients with T1DM	PTA	Not specified	MNSI, NCS, VPT	Improvement in MNSI, peroneal amplitude, and VPT
Kluding et al. 2012	Pre–post test (single arm)	17 patients with DPN	Aerobic and resistance exercise	10 weeks	Pain (VAS), MNSI, nerve function, IENF density/branching	Reduction in pain and MNSI scores, with increased IENF branching
Singleton et al. 2014	Randomised controlled trial	100 patients with T2DM without DPN (60 supervised weekly exercise vs. lifestyle counselling)	Supervised weekly exercise	12 months	IENFD, UENS, NCS	Improvement in IENFD
Reynolds et al. 2023	Prospective cohort study	127 patients with Class II/III obesity (79 completed follow‐up)	Bariatric surgery	2 years	IENFD, NCS, MNSI, QST and UENS	Improvement in IENFD
Casellini et al. 2024	Randomised controlled trial (Phase II)	51 patients with T2DM and confirmed DPN (27 oxybutynin vs. 24 placebo)	Topical oxybutynin	20 weeks	IENFD, pain scores, clinical scales, NCS, CCM	Improvement in IENFD, pain scores and quality of life

Abbreviations: ATTRv, hereditary transthyretin amyloidosis; CCM, corneal confocal microscopy; CNFD, corneal nerve fibre density; CNFL, corneal nerve fibre length; DPN, diabetic peripheral neuropathy; IENFD, intraepidermal nerve fibre density; mNIS+7, modified Neuropathy Impairment Score plus 7 neurophysiological tests; MNSI, Michigan Neuropathy Screening Instrument; NCS, nerve conduction studies; NCV, nerve conduction velocity; PTA, pancreas transplant alone; QoL, quality of life; QOL‐DN, Norfolk Quality of Life‐Diabetic Neuropathy Questionnaire; RCT, randomised controlled trial; SPK, simultaneous pancreas–kidney transplant; T1DM, Type 1 diabetes mellitus; T2DM, Type 2 diabetes mellitus; UENS, Utah Early Neuropathy Scale; VAS, Visual Analogue Scale; VPT, vibration perception threshold.

### 2.1 Hereditary Transthyretin Amyloidosis, an Instructive Case for DPN

Before examining the current landscape for DPN, it is instructive to consider ATTRv, in which recent therapeutic advances have demonstrated that clinical peripheral neuropathy can be reversed [13]. ATTRv is an autosomal dominant, adult‐onset systemic disease caused by mutations in the gene encoding transthyretin (TTR). This mutation leads to TTR misfolding and aggregation, forming amyloid fibrils that deposit and accumulate in multiple organ systems. When this deposition involves the peripheral nervous system, it produces a progressive neuropathy, with median survival ranging from 3 to 15 years after onset [14].

For two decades, the only therapies with substantial clinical benefit were liver transplantation (or combined heart–liver transplantation), which aimed to suppress the main source of circulating TTR. However, continued deposition of amyloid and progression of disease post‐transplant remained ongoing challenges. In recent years, substantial progress has been achieved in the treatment of ATTRv with the development of novel therapies such as gene‐silencing therapies, which suppress hepatic TTR production [15, 16]. Inotersen is an antisense oligonucleotide that reduces the levels of TTR transcript, while patisiran is an RNA interference agent that inhibits TTR production. Both agents have shown efficacy in clinical trials using the modified Neuropathy Impairment Score+7 (mNIS+7), a robust composite outcome measure incorporating motor, sensory, autonomic and neurophysiological assessments [17]. Inotersen significantly slowed the progression of neuropathic impairment compared with placebo, with 36% of participants demonstrating improvements in mNIS+7 from baseline [15]. In contrast, patisiran not only prevented progression but led to mean improvements in mNIS+7, with 56% of participants showing improvement from baseline [16]. Importantly, both trials also demonstrated benefits in quality of life, assessed using the Norfolk Quality of Life‐Diabetic Neuropathy Questionnaire [15, 16].

Although ATTRv and DPN differ substantially in causation, both are length‐dependent polyneuropathies with potential small‐fibre involvement early and more widespread axonal sensory‐motor dysfunction later [18]. We therefore cite ATTRv not as a mechanistic parallel to DPN, but as a clinical proof‐of‐principle that peripheral neuropathy may retain some capacity for improvement when its dominant pathogenic driver is effectively addressed. In this sense, ATTRv provides a useful example of how disease‐modifying therapy can alter the natural history of an established neuropathy, even if the underlying biology differs from that of DPN.

### 2.2 Pancreatic Transplantation

In Type 1 diabetes mellitus (T1DM), autoimmune destruction of pancreatic β cells lead to insulin deficiency. PT remains the most effective treatment to replace insulin‐producing cells, producing dramatic and durable improvements in glycaemic control and, in many cases, insulin independence.

Between 1989 and 2023, 16 articles investigated the impact of PT (with or without simultaneous renal transplant, SPK) on DPN [8, 19, 20, 21]. These studies have employed a range of metrics to measure neuropathy, including nerve conduction studies (NCSs), vibration perception threshold, corneal confocal microscopy (CCM) and clinical scoring systems (e.g., Michigan Neuropathy Screening Instrument [MNSI]) [19]. Of these, 14 studies reported improvements in neuropathy outcomes, including in NCS, a confirmatory test for large fibre involvement. For example, a retrospective case–control study comparing 36 individuals with T1DM undergoing SPK with 29 controls found significant improvements in CCM measures and peroneal nerve conduction velocity and amplitude in the transplant group [8]. Although the overall quality of evidence is limited, largely consisting of small‐ to medium‐sized retrospective studies, the findings consistently suggest that PT may partially reverse objective measures of neuropathy in T1DM.

### 2.3 Bariatric Surgery and Lifestyle Intervention for Type 2 Diabetes

Despite recent advances in therapeutics for Type 2 diabetes mellitus (T2DM), there is no equivalent to PT in T1DM for restoring metabolic control. However, lifestyle interventions and bariatric surgery have shown promising effects in DPN.

Kluding et al. studied 17 individuals with T1DM and DPN who participated in a 10‐week aerobic and strengthening programme, reporting improvements in both MNSI and intraepidermal nerve fibre density (IENFD) [22]. Similarly, Smith et al. found improvements in IENFD in 32 people with pre‐diabetes following a structured diet and exercise intervention [23]. While Singleton et al. studied 100 participants with T2DM without DPN, showing that supervised weekly exercise for 1 year (*n* = 60) produced improvements in IENFD compared with quarterly lifestyle counselling (*n* = 40) [24]. Stubbs et al. (*n* = 45) found that in veterans with long‐standing Type 2 diabetes and established DSPN, 12 weeks of structured exercise did not improve standard nerve conduction outcomes overall, but some participants showed improved IENFD [25].

Bariatric surgery, a more effective but intensive intervention for T2DM and obesity, has been associated with improvements in peripheral neuropathy. In a prospective cohort study of 127 individuals with Class II/III obesity (30% normoglycaemia, 41% pre‐diabetes, 29% diabetes), assessments at 2 years showed improvement in one out of two primary outcomes (IENFD at the thigh), two secondary outcomes, and several patient‐reported measures [26]. Although the evidence for bariatric surgery/lifestyle intervention remains limited, largely from small, non‐randomised or observational studies, these findings suggest that reversal of metabolic dysfunction may promote nerve regeneration.

### 2.4 Oxybutynin as a Promising Agent for DPN Reversal

Despite advances, no pharmacological therapy has proven a disease‐modifying effect in DPN. However, muscarinic receptor antagonists are emerging candidates [27]. Pre‐clinical studies have shown that muscarinic antagonists promote mitochondrial function and induce axonal repair in various neuropathies in rodent models [27, 28].

One randomised, double‐blind, placebo‐controlled trial of 3% oxybutynin gel has been performed in 51 people with T2DM and DPN [28]. The primary endpoint was the within‐group change from baseline IENFD at the proximal leg after 20 weeks of treatment. IENFD significantly increased in the oxybutynin‐treated group, with no significant difference in the placebo group, confirmed by two independent pathology reviewers. In addition, other clinical measures of neuropathy improved with oxybutynin, including clinical examination scoring systems, symptoms and quality of life.

This proof‐of‐concept trial indicates that muscarinic antagonists may be a novel therapeutic agent for DPN. Moreover, this trial offers further evidence that clinical DPN is potentially reversible, but confirmation is required in larger randomised controlled trials (RCTs).

### 2.5 Conclusion

**FIGURE 1:**
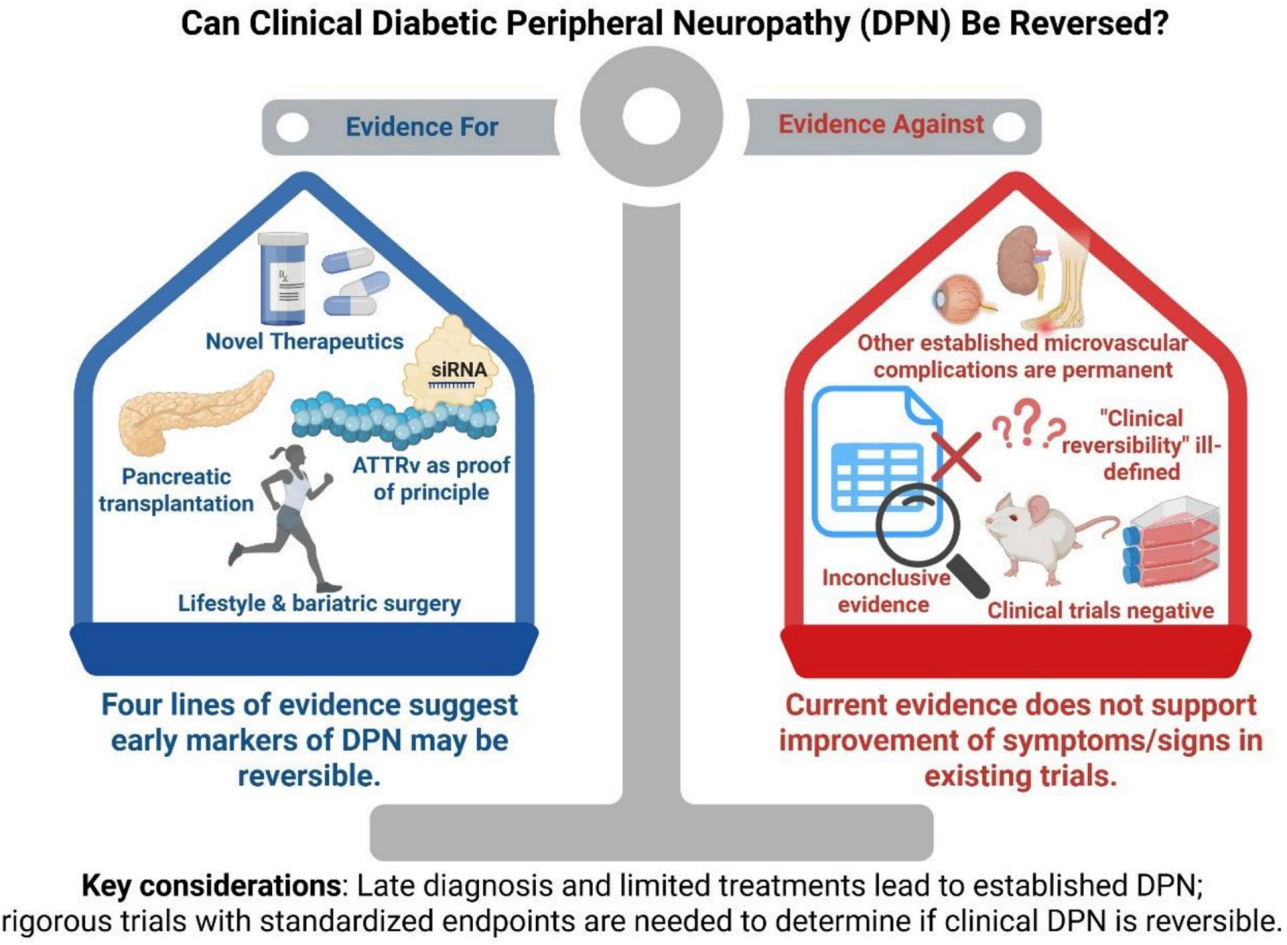
Can clinical diabetic peripheral neuropathy (DPN) be reversed?

Taken together, these four lines of evidence demonstrate that DPN is not inevitably progressive and irreversible (Table 1 and Figure 1). Lessons from ATTRv confirm that targeted therapies can restore nerve function; PT shows that disease‐modifying interventions in diabetes can improve neuropathy; lifestyle and bariatric surgery demonstrate that metabolic correction supports nerve regeneration; and novel agents such as oxybutynin highlight therapeutic potential. While the strength of evidence varies, collectively these data support the position that clinical DPN can be reversed.

## 3 The Case *Against* Clinical DPN Being Reversible

The case against clinical DPN being reversible will consist of four main arguments (Table 2): (1) By analogy, other microvascular complications of diabetes do not reverse at clinical stages; (2) defining ‘reversibility’ is complicated and impedes obtaining evidence; (3) systematic evidence for clinical reversal—rather than numerical improvement in surrogate measures—is lacking; (4) promising therapies in pre‐clinical studies have not translated to efficacy in human trials.

**TABLE 2:** Summary of key clinical studies reporting no reversal of diabetic peripheral neuropathy.

Study	Evidence level	Sample	Active intervention	Follow‐up	Outcome metrics	Results
Partanen et al. 1995	Prospective cohort study	133 patients with T2DM	N/A^a^	10 years	NCV, VPT, symptoms	Progression in neuropathy despite modest metabolic control
DCCT Study Group 1993	Randomised controlled trial	1441 patients with T1DM (730 conventional vs. 711 intensive glucose control)	Intensive glucose control	6.5 years	Neuropathy incidence	Reduction in DPN incidence, no reversal in existing cases
Albers et al. 2010	Long‐term observational follow‐up of DCCT cohort	1186 patients with T1DM (583 conventional vs. 603 intensive glucose control)	Prior intensive vs. glucose control	13–14 years post‐trial	Clinical exam, NCS	Prior intensive therapy reduced incidence, but existing neuropathy progressed; no evidence of reversal
Akbari et al. 1997	Observational cohort	55 patients with DM requiring lower‐extremity arterial bypass	Lower‐extremity arterial reconstruction	19.2 months	NCV	NCV remained stable post‐op; progression halted in revascularised limb, worsened in control limb; no reversal observed
Pop‐Busui et al. 2013	Post hoc analysis of randomised controlled trial	2159 patients with T2DM and CAD (1080 insulin‐sensitising vs. 1079 insulin‐providing)	Insulin‐sensitising	4 years	MNSI	Incidence of DPN lower with insulin‐sensitising. No reversal in established DPN
Navarro et al. 1997	Prospective cohort study	115 patients with PT vs. 92 insulin‐treated controls	PT	10‐year follow‐up	Clinical examination score, NCS	NCS showed significant improvements; however, minimal or no meaningful clinical change
UKPDS 1998	Randomised controlled trial	3867 patients with T2DM (2729 intensive treatment vs. 1138 conventional)	Intensive glucose control with sulphonylurea or insulin	10 years	Tendon reflexes, abnormal VPT	No evidence that intensive glucose control altered neuropathy outcomes
Ziegler et al. 2011	Randomised controlled trial	460 patients with mild‐to‐moderate DPN (233 α‐lipoic acid vs. 227 placebo)	α‐Lipoic acid	4 years	NIS(LL)+7	No difference between active treatment and placebo arm
Malik et al. 1998	Randomised controlled trial	46 patients with mild DPN (23 tradalopril vs. 23 placebo)	Tradalopril	12 months	NCS, symptoms, VPT, examination score	Significant but clinically mild improvement in NCS measures, not in symptoms VPT or examination scores

Abbreviations: DPN, diabetic peripheral neuropathy; MNSI, Michigan Neuropathy Screening Instrument; NCV, nerve conduction velocity; NIDDM, non‐insulin‐dependent diabetes mellitus; NIS(LL)+7, Neuropathy Impairment Score of the Lower Limb plus 7 neurophysiological tests; PT, pancreatic transplantation; PVD, peripheral vascular disease; RCT, randomised controlled trial; T1DM, Type 1 diabetes mellitus; T2DM, Type 2 diabetes mellitus; VPT, vibration perception threshold.

^a^ No intervention, natural history observation.

### 3.1 Other Microvascular Complications of Diabetes Do Not Reverse at Clinical Stages

DPN is one of the three complications of Diabetes commonly referred to as ‘microvascular complications’, alongside nephropathy and retinopathy. In nephropathy, the earliest detectable marker is microalbuminuria, which may regress if shorter in duration and if modifiable risk factors such as glycaemia, blood pressure and lipids are controlled, but improvement in renal function by estimated glomerular filtration rate has not been systematically observed [29, 30, 31, 32]. Similarly, diabetic retinopathy at its earlier stages may regress, particularly in the presence of lower glucose and serum triglyceride levels [33].

However, once microvascular complications are established in clinical stages in the presence of clinically evident end‐organ structural damage, progression can only be slowed, not reversed. In T1DM, PT improves the histological changes on renal biopsies, yet creatinine clearance continued to decline, not improve [34]. Similarly, in T2DM, sodium glucose transport‐2 inhibitors (SGLT2i) slow the decline in estimated glomerular filtration rate but do not restore renal function [35]. Thus, while early microvascular disease is dynamic, established clinical stages of disease are not strictly reversible. Given the well‐established clinical irreversibility of other microvascular complications, there is no substantive or principled basis to expect that DPN would exhibit a reversible trajectory.

### 3.2 A Definition of ‘Reversibility’ for Clinical DPN Is Complicated and Impedes Obtaining Evidence

A major barrier to claims of reversibility in DPN lies in the inherent complexity of its presentation and definition. Although often described as progressing from early nerve dysfunction to complications, this trajectory of DPN is neither linear nor uniform. DPN comprises heterogeneous phenotypes: some individuals experience neuropathic pain; others develop numbness or imbalance and some remain asymptomatic despite significant neuronal dysfunction. Apparent ‘improvement’ may therefore reflect a shift in phenotype rather than genuine recovery of nerve function.

From a diagnostic perspective, ‘confirmed’ clinical DPN requires symptom(s) and/or sign(s) and objective abnormalities of nerve function [7, 36]. NCSs are the most widely used confirmatory test for large fibre abnormalities (and IENFD for small fibre). Yet diagnostic thresholds of NCS vary by region, and even diagnosing DPN represents a continuum of probability based on a combination of symptoms, signs and test results [36]. Within this framework, the concept of ‘reversal’ is inherently ambiguous. Resolution of symptoms may accompany progressive sensory loss; for example, disappearance of pain due to complete loss of small‐fibre function. Symptoms and objective measures of neuropathy represent distinct constructs, and improvement in any single domain cannot be construed as definitive evidence of true disease reversal. Finally, an objective and measurable outcome of DPN could be ulceration or amputation [37]; and although DPN is the strongest risk factor for both, vascular disease, deformity and foot care influence amputation rates [38]. Thus, reductions in these complications alone cannot be taken as proof of neuropathy reversal.

In summary, the complexity and heterogeneity of DPN diagnosis, presentations, and natural history trajectory create difficulties in establishing what constitutes reversal, and without a consistent, universally accepted definition, evidence generation for reversibility remains complicated.

### 3.3 Systematic Observational Cohort or Trial Evidence for Clinical Remission Is Certainly Lacking

The earliest evidence for DPN suggested that it is a condition which progressively worsens with time, with poorer glycaemic control predicting deterioration [39]. Observational studies show that objective measures of DPN continue to decline, even when metabolic control is improved [40]. Moreover, more intensive interventions such as lower‐extremity arterial reconstruction are able to halt DPN progression, but do not induce reversal [41].

The Diabetes Control and Complications Trial (DCCT), and subsequent long‐term follow‐up in Epidemiology of Diabetes Interventions and Complications (EDIC) [42], provided the strongest evidence that early and sustained intensive insulin therapy reduces the incidence and progression of DPN in T1DM, but it did not demonstrate reversal of established neuropathy [43, 44]. Similarly, in T2DM, the BARI 2D trial reported changes in clinical scoring ‘remission’ of DPN with both insulin‐providing and insulin‐sensitising treatments, but the study lacked robust structural or neurophysiological endpoints [45]. Therefore, as discussed above, shifts in clinical scores may reflect changes in phenotype rather than true remission. Even the strongest interventions do not provide convincing evidence of reversal. In individuals receiving PT compared with controls, deterioration was slower, but improvements were modest overall [46]. More recent SPK studies demonstrate gains in CCM and some neurophysiological measures, as mentioned above, to support evidence of clinical reversal, but these reflect attenuation or partial improvement rather than restoration of nerve function to normal levels. While CCM represents an outstanding opportunity to track biological response to therapies targeting small nerve fibre morphology, it is only a surrogate measure and not a clinical measure of neuropathy.

Finally, RCTs introduce additional complexity: placebo groups have shown improvements in surrogate neurophysiological measures, often associated with glycaemic control and lipid levels, raising the question of whether observed changes represent true treatment effects or reflect natural fluctuations during DPN [47, 48, 49]. However, regardless of these issues, the strongest causal evidence is observed in an RCT of intensive lifestyle intervention (the ‘Look AHEAD’ trial), which reports extremely subtle improvements in symptoms and a selected sign certainly without effects on clinical outcomes in keeping with clinical reversal of neuropathy [50].

### 3.4 Promising Therapies in Pre‐Clinical Studies Have Not Translated Into Efficacy in Human Trials

Despite decades of promising pre‐clinical findings, no disease‐modifying therapy has succeeded in reversing clinical neuropathy in humans. High‐certainty evidence exists for the lack of multiple therapeutic classes targeting metabolic, oxidative, inflammatory and vascular pathways [51]. Low‐certainty evidence supports modest effects of lifestyle interventions [23], angiotensin converting enzyme inhibitors [52], lipid‐lowering therapy [53] and α‐lipoic acid [54]. The strongest evidence remains for intensive blood glucose control, whether using intensive insulin therapy [43, 55] or PT [46]. However, at best, the evidence for efficacy is based on improvement on surrogate markers, or reducing incidences/progression of disease, but not reversal of clinical DPN.

Even the recent oxybutynin trial, cited as evidence ‘for’, showed no significant improvement in the Neuropathy Impairment Score in the Lower Limbs, an objective clinical endpoint, despite improvements in IENFD [28]. While this antimuscarinic therapeutic class holds major promise in view of these surrogate findings, the current evidence underscores the gap between surrogate marker improvements and meaningful clinical reversal.

### 3.5 Conclusion

Taken together, the evidence argues strongly against clinical DPN being reversible (Table 2 and Figure 1). Early microvascular complications may regress, but once established at clinical stages, progression can only be slowed. The heterogeneity of DPN and the lack of a clear definition of reversal complicate interpretation. Landmark trials and long‐term interventions demonstrate attenuation, not reversal. Finally, repeated failures of promising therapies in clinical trials underscore the challenge. Collectively, these data support the position that clinical DPN is not reversible.

## 4 Discussion

DPN remains one of the most common and debilitating complications of diabetes, associated with pain, sensory loss, increased risk of foot ulceration, amputation and reduced quality of life. The debate on whether clinical DPN is reversible is therefore of major clinical and scientific importance, as it directly informs research priorities and therapeutic strategies (Figure 1).

Defining ‘reversibility’ in DPN is complicated. The heterogeneity of DPN phenotypes, variability in diagnostic thresholds and lack of agreement on outcome measures mean that there is no agreed‐upon definition of reversibility. Greater consistency in both the definition of DPN and the outcome measures used in clinical trials is urgently needed [56]. A recent international expert consensus statement highlighted the importance of establishing meaningful, valid, and standardised outcomes for DPN intervention studies. Moreover, patient‐centred measures (e.g., quality of life) and hard clinical outcomes (e.g., amputation and mortality) should also be incorporated wherever feasible. Expert opinion aside, a general clinical trialist would insist that a meaningful clinical outcome that could lead to drug approval would need to be based on patient clinical experience as opposed to surrogate measurements such as NCS, CCM or IENFD.

The distinction between *remission* and *reversibility* in DPN is clinically important yet often overlooked in the interpretation of therapeutic outcomes. *Reversibility* implies restoration of normal nerve structure and function through regeneration of damaged axons, reinnervation of target tissues and recovery of physiological sensory and autonomic function, which appears unlikely in the context of clinically established disease. By contrast, *remission* refers to a state of symptomatic or functional improvement without necessarily achieving normalisation. Individuals with DPN may experience reduced pain, improved sensory thresholds, or gains in surrogate measures such as CCM or IENFD, while residual or subclinical neuropathic deficits persist. This distinction parallels that in other microvascular complications of diabetes. In diabetic retinopathy and nephropathy, early‐stage regression or ‘remission’ can occur with improved metabolic control, but once advanced structural damage such as proliferative retinopathy or glomerulosclerosis is established, full *reversibility* remains elusive [57]. Similarly, in DPN, stabilisation or partial recovery of small‐fibre measures may reflect remission rather than full reversibility. Long‐term observational studies and large randomised trials, such as DCCT/EDIC, showed clear benefits of intensive glycaemic control in preventing or delaying DPN onset, but not reversal of established disease [58]. In addition, long‐term studies of PT demonstrate attenuation of decline and modest improvements in electrophysiological measures, but not restoration to normality [19, 29]. This disconnect between surrogate marker gains and functional recovery underscores the challenge of translating experimental promise into meaningful clinical reversal and highlights the need for clear and standardised definitions of ‘remission’ and ‘reversibility’ in DPN to enable consistent interpretation of therapeutic outcomes.

Beyond the conceptual distinction between remission and reversibility, therapeutic interpretation should also consider the magnitude of treatment effect, which is essential for contextualising outcomes in DPN. Many signals in DPN trials represent small effects, which are statistically significant but of limited clinical relevance, paralleling the modest benefits seen with cholinesterase inhibitors in Alzheimer's disease [59]. What is required for DPN are large‐effect interventions, which mirror the transformative impact of inotersen and patisiran for ATTRv amyloidosis [13, 16]. Outside of therapies for neurological disease, GLP‐1 receptor agonists for weight reduction and statins for cardiovascular risk reduction further illustrate what large‐effect therapies can achieve. By contrast, existing DPN treatments have so far delivered only small effects, rather than true reversal.

Despite decades of translational research, no disease‐modifying therapies are currently established for DPN, although optimisation of cardiovascular risk factors, including hypertension [52, 60] and dyslipidaemia [53, 61, 62], may reduce onset or provide benefit in early disease. Timing appears to be a critical determinant of therapeutic potential, as early neuropathy is largely functional and potentially reversible, whereas prolonged metabolic injury leads to structural damage that is unlikely to be reversed, shifting the goal towards remission rather than recovery. Moreover, numerous candidate therapies, including neurotrophins, aldose reductase inhibitors, antioxidants and gene‐based approaches, have shown promise in pre‐clinical models but consistently failed to modify disease progression in clinical trials [6], suggesting that these represent true therapeutic failures rather than limitations of study design, and highlighting the substantial biological barriers to reversing established DPN.

The arguments highlight both optimism and caution. The case for reversibility is strengthened by emerging data across transplantation, lifestyle and pharmacological domains, alongside analogies from non‐diabetic neuropathies. Yet, the opposing view emphasises the weight of historical evidence, the limitations of current interventions, and the unresolved complexity in defining and measuring reversal.

This debate highlights a central tension in the field of DPN. The expert audience voted in favour of the motion (with an approximate 60:40 split), that ‘This house believes that clinical diabetic peripheral neuropathy is reversible’; although ultimately, the question of whether clinical DPN is reversible may depend less on absolute possibility and more on timing, definition, and measurable endpoints. Reversal is likely confined to early stages, but once advanced structural nerve damage is established, complete regeneration may be impossible. Future research should prioritise the identification of biomarkers of early neuronal dysfunction, the refinement of outcome measures and the development of combination strategies integrating metabolic optimisation, regenerative and neuroprotective approaches to shift DPN from an irreversible to a modifiable disease state. Thus, the concept of reversibility may be best viewed not as a binary outcome, but as a continuum reflecting the biological potential for regeneration within the practical constraints of clinical intervention.

## Data Availability

Data sharing not applicable to this article as no datasets were generated or analysed during the current study.
